# Metabolomic data of melittin-intervened murine cervical cancer cells based on liquid chromatography-mass spectrometry

**DOI:** 10.1016/j.dib.2026.113051

**Published:** 2026-07-02

**Authors:** Jianrong Jiang, Yaqin Gao, Mengyi Wang, Ronghua Zhang, Jiwu Lou, Yueliang Li, Jianfeng Qiu, Dafu Chen, Tizhen Yan, Rui Guo, Yanhui Liu

**Affiliations:** a Dongguan Maternal and Children Health Hospital, Dongguan, 523000, China; b Dongguan Technology Transfer Centre of National & Local United Engineering Laboratory of Natural Biotoxin, Dongguan, 523000, China; c National & Local United Engineering Laboratory of Natural Biotoxin, Fuzhou, 350002, China; d Apitherapy Research Institute of Fujian Agriculture and Forestry University, Fuzhou, 350002, China; e Shenzhen Luohu People’s Hospital, Shenzhen 518000, China

**Keywords:** Melittin, Cervical cancer, U14 cells, Metabolomics, Metabolites

## Abstract

Melittin-treated murine cervical cancer U14 cells have been widely recognized as a classic cellular model for anti-tumor research in cervical cancer. This article contains metabolomic data of U14 cell lysates from both melittin-treated and control groups. Untargeted metabolomic profiling was carried out by liquid chromatography-mass spectrometry (LC-MS) to systematically elucidate the global metabolic disturbances in cervical cancer cells upon melittin intervention. LC-MS raw data were processed for peak extraction and alignment using XCMS software, followed by quality control normalization with metaX software. Metabolite annotation was performed against the HMDB and KEGG databases as well as an in-house MS/MS spectral library, yielding metabolite feature data including mass-to-charge ratio (*m/z*), retention time (RT), and MS/MS-identified metabolites (MS2). A total of 22,976 metabolic ions were detected in this study, among which 16,176 were assigned Level 1 annotations and 1114 were identified with high confidence at Level 2. All raw and processed data are publicly accessible at NGDC (accession number PRJCA065444). This untargeted LC-MS-based metabolomic dataset not only provides a comprehensive resource for elucidating metabolism-related anticancer mechanisms of melittin in murine U14 cervical cancer cells but also supports the development of targeted therapeutic strategies against cervical cancer.

Specifications Table

**Table:** 

Subject	Biology
Specific subject area	Untargeted LC-MS-based metabolomic profiling of melittin-treated murine U14 cervical cancer cells.
Type of data	Raw
Data collection	U14 cells were divided into melittin-treated and untreated group, with five biological replicates per group. After sample collection, metabolite extraction was performed, and the extracts were subjected to LC-MS analysis in both positive and negative ion modes.
Data source location	Data were collected at the Apitherapy Research Institute, College of Bee Science, Fujian Agriculture and Forestry University (Fuzhou, China) and the Dongguan Technology Transfer Center of National & Local United Engineering Laboratory of Natural Biotoxin (Dongguan, China). Metabolomic analyses were performed at these institutions. All raw and processed data are stored at the authors' affiliations: Fujian Agriculture and Forestry University and Dongguan Maternal and Children Health Hospital.
Data accessibility	Repository name: NGDC (National Genomics Data Center) Genome Sequence Archive Data identification number: Database BioProject: PRJCA065444 Database OMIX: OMIX017279 Direct URL to data: Database BioProject: https://ngdc.cncb.ac.cn/bioproject/browse/PRJCA065444 Database OMIX: https://ngdc.cncb.ac.cn/omix/release/OMIX017279 Instructions for accessing these data: The dataset can be accessed through the NGDC website by searching with the BioProject accession number PRJCA065444. Reviewers may use this link for anonymous access during the review process.
Related research article	None.

## 1 Value of the Data

• Both the intracellular metabolic profiles of melittin and untreated U14 cells were obtained, offering an overview of the regulatory effect of melittin on the global metabolism of cervical cancer cells.
• The datasets are beneficial for researchers working on the anti-cervical cancer mechanisms of melittin and natural bioactive compounds, as well as metabolic studies in cellular models.
• Our data will facilitate the understanding of the metabolic characteristics of the cervical cancer cell model, cervical cancer pathogenesis, and drug intervention mechanisms.

## 2 Background

Murine U14 cervical cancer cells are a classic cervical cancer research model possessing both *in vitro* culture and *in vivo* tumorigenic capabilities [[1], [2], [3]]. Melittin, the principal active peptide of bee venom, demonstrates pronounced biological activities, notably the inhibition of cancer cell proliferation and the induction of apoptosis, and has consequently attracted considerable attention in the field of natural antitumor drug discovery [[4], [5], [6]]. Despite these findings, the global metabolic alterations underlying melittin’s anti-cervical cancer effects remain largely unexplored. To address this gap, an untargeted metabolomic approach based on liquid chromatography-mass spectrometry (LC-MS) was employed to profile the intracellular metabolite changes in U14 cells following melittin exposure compared with vehicle-treated controls. The data presented here provide a valuable resource for understanding the metabolic reprogramming induced by melittin and may facilitate the identification of potential pharmacodynamic biomarkers and therapeutic targets.

## 3 Data Description

This article contains all metabolomic analysis data obtained from melittin-treated (T) and vehicle control (C) U14 cell samples using liquid chromatography-mass spectrometry (LC-MS). The datasets containing metabolite name, database ID, mass-to-charge ratio (*m/z*), retention time (RT), matched Level 2 metabolite name, and matched database ID are provided (Table 1); the relative quantification data of secondary metabolites are also provided (Table 2).

**Table 1:** Processed metabolomic identification data of U14 cervical cancer cells.

ID	MZ	RT	MS2.name	MS2.score	HMDB	KEGG
NEG_307.2268_579.4786_1	307.23	579.48	1,1,1-Trifluoroheptadecan-2-one	1.00	HMDB0256073	NA
POS_98.9844_39.8767_1	98.98	39.88	1,1-Dichloroethane	0.94	HMDB0244020	C18247
NEG_311.1676_441.1647_1	311.17	441.16	1,2,3,4-Tetrahydro-1-phenyl-4-(1-phenylethyl)naphthalene	0.96	HMDB0040520	NA
NEG_96.9647_41.1657_1	96.96	41.17	1,2-Dichloroethane	0.97	HMDB0029571	C06752
NEG_211.1326_449.1033_1	211.13	449.10	1,4′-Bipiperidine-1′-carboxylic acid	0.86	HMDB0060336	C16836
POS_185.1291_331.8651_1	185.13	331.87	1,4-Dimethyl-7-ethylazulene	0.90	HMDB0036470	C09633
NEG_421.2252_463.5894_1	421.23	463.59	1,4-Dioctyl sulfosuccinic acid	0.85	HMDB0251779	NA
POS_160.0762_281.701_1	160.08	281.70	1,N6-Ethenoadenine	0.94	HMDB0244259	NA
POS_220.1186_164.8592_1	220.12	164.86	1-(2,3-Dihydro-6-methyl-1H-pyrrolizin-5-yl)-1,4-pentanedione	0.88	HMDB0040006	NA
POS_279.2051_353.5818_1	279.21	353.58	1-(4-Hydroxy-3-methoxyphenyl)-3-decanone	0.85	HMDB0030801	C10482

MS2.= MS/MS-identified metabolites.

**Table 2:** HMDB superclass annotation of metabolites in U14 cervical cancer cells.

SuperClass	Metabolites_nums	MS2.name
Lipids and lipid-like molecules	370	6-methylhexadecanoic acid
Organoheterocyclic compounds	204	Adenine
Organic acids and derivatives	158	Glycolic acid
Benzenoids	138	4-Nitrophenol
Organic oxygen compounds	56	Malondialdehyde
Organic nitrogen compounds	47	Guanidinopropionic acid
Phenylpropanoids and polyketides	47	Glycitin
Nucleosides, nucleotides, and analogues	44	N6-Methyladenosine
Alkaloids and derivatives	15	Corynanthine
Organosulfur compounds	13	Dimethyl sulfoxide

MS2.= MS/MS-identified metabolites.

## 4 Experimental Design, Materials and Methods

### 4.1 Cell culture and melittin treatment

**Fig. 1:**
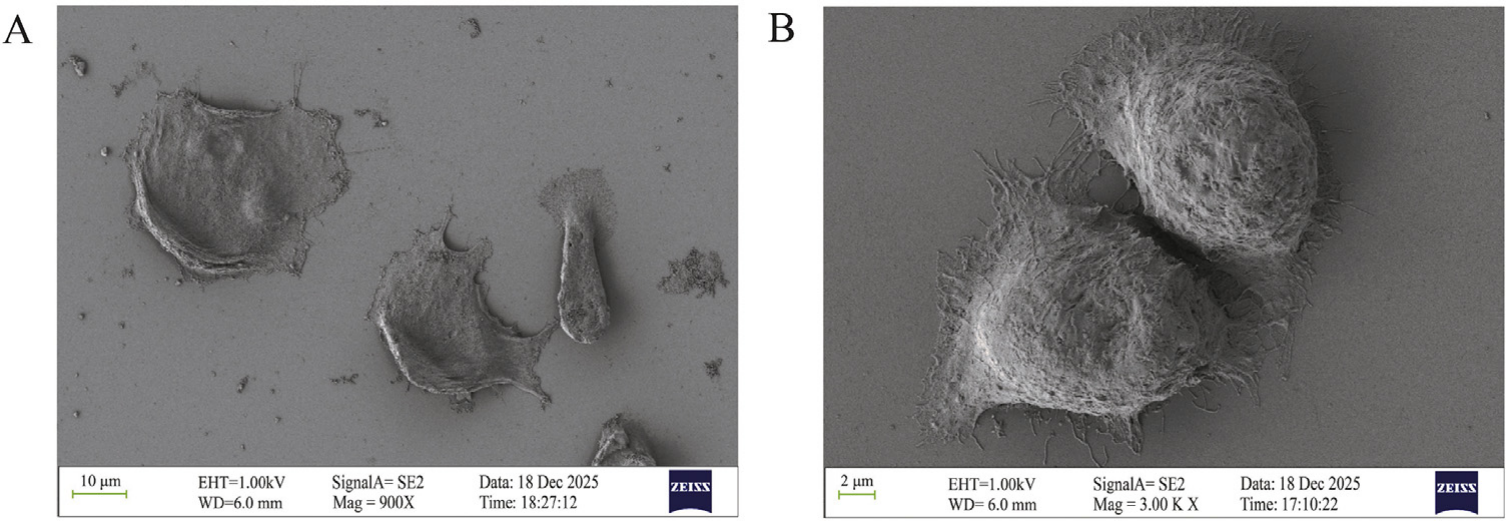
Scanning electron microscopy (SEM) images of murine cervical cancer U14 cells. A. Vehicle control group. B. Melittin-treated group. EHT: Extra High Tension. WD: Working Distance. SE2: Secondary Electron 2. Mag: Magnification.

Murine cervical cancer U14 cells were routinely cultured in DMEM (Dulbecco's Modified Eagle Medium) high glucose medium supplemented with 10% fetal bovine serum (FBS) and 1% penicillin-streptomycin, and maintained in a humidified incubator at 37 °C with 5% CO₂. To ensure that cells were in the logarithmic growth phase for all experiments, a preliminary growth curve was established: cells were seeded in 24-well plates at a density of 1 × 10⁴ cells/well, and viable cells were counted daily using a hemocytometer for 7 consecutive days, followed by definition of the logarithmic (exponential) phase as the linear portion of the semi-logarithmic plot of cell number *versus* time (population doubling time approximately 24 h). Based on this curve, cells at 48–72 h after seeding were confirmed to be in the logarithmic phase and then used in subsequent experiments. Cells in the logarithmic growth phase were harvested and divided into a vehicle control group (C) (Fig. 1-A) and a melittin-treated group (T) (Fig. 1-B), with five biological replicates per group. After treatment, cell samples were collected and then subjected to metabolite extraction.

### 4.2 Metabolite extraction and quality control preparation

By using an organic solvent precipitation method [7], cell samples were collected and small-molecule metabolites were then extracted. Quality control (QC) samples were prepared through pooling equal aliquots of all experimental samples and then injected before and after LC-MS detection as well as interspersed throughout the analytical sequence to monitor instrument stability and data reproducibility.

### 4.3 Metabolomic profiling

Untargeted metabolomic profiling was performed on a liquid chromatography-mass spectrometry (LC-MS) platform in both positive and negative ion modes. Raw mass spectrometric data were acquired, including *m/z*, retention time, and peak intensity for each metabolic ion.

### 4.4 Data processing and statistical analysis

The acquired MS data were pretreated with XCMS software for peak picking, peak alignment, and signal correction. Data quality control was conducted with metaX software, where low-quality signals were filtered out, and data were further preprocessed by median normalization and missing value imputation. Metabolites were annotated by matching accurate precursor masses and MS/MS fragmentation spectra against the HMDB (https://hmdb.ca/), KEGG (https://www.kegg.jp/), and an in-house database. Differential metabolites between groups were identified using Student’s *t*-test combined with variable importance in projection (VIP) values from a PLS-DA model. Screening criteria were set as fold change (FC) ≥ 1.2 or ≤ 1/1.2, P < 0.05, and VIP ≥ 1.

## Limitations

Although this study reports the first comprehensive untargeted metabolomic dataset of melittin-treated murine U14 cells, the metabolic profiling is generated entirely from an *in vitro* cellular model. Therefore, it only provides initial information on the global metabolic disturbances induced by melittin in cervical cancer cells. More *in vivo* animal data and targeted metabolomic analyses should be explored to provide sufficient data for elucidating the systemic therapeutic mechanisms, precise metabolic biomarkers, and targeted therapeutic strategies of melittin in treating cervical cancer.

## Ethics Statement

The authors have read and follow the ethical requirements for publication in Data in Brief. The current work does not involve human subjects, animal experiments, or any data collected from social media platforms. All experiments were conducted using a commercially available murine cervical cancer cell line (U14), which was obtained from Xiamen Yimo Biotechnology Co., Ltd. No human or animal subjects were used in this study.

## CRediT Author Statement

Jianrong Jiang Investigation, Writing-original draft. Yaqin Gao Formal analysis, Writing-original draft. Mengyi Wang, Ronghua Zhang and Jiwu Lou Investigation and Resources. Yueliang Li, Jianfeng Qiu, and Dafu Chen Resources. Tizhen Yan and Rui Guo Writing-review & editing, Project administration and Supervision. Yanhui Liu, Writing-review & editing and Supervision. All authors read and approved the final manuscript.

## Data Availability

NGDCLC-MS Metabolomics of Melittin-Treated Murine Cervical Cancer Cells (Original data)
